# The Cost of Lost Follow-Up: A Diagnosis of Sarcomatoid Lung Carcinoma Following a Presumed Benign Pulmonary Mass and the Necessity of a Physician's Suspicion

**DOI:** 10.7759/cureus.94148

**Published:** 2025-10-08

**Authors:** Xochitl A Moreno Gonzalez, Peri G Mcclaskey, Michelle K Vu, Hamid Gogerdchian, Sanjana Murdande, Kevin T Dao, Alaleh Bazmi

**Affiliations:** 1 Internal Medicine, University of California, Los Angeles (UCLA) Kern Medical, Bakersfield, USA

**Keywords:** clinical management, false negative, high clinical suspicion, lost follow-up, pulmonary mass, sarcomatoid lung carcinoma

## Abstract

Pulmonary sarcomatoid carcinoma is a rare non-small cell lung cancer that can rapidly progress and, unfortunately, serves as a diagnostic challenge. In many instances, patients can have non-specific symptoms, and the malignancy itself can produce negative biopsy results, leading to a false sense of security. This is especially true in larger tumors, since surrounding inflammation can obscure malignant tissue. As such, situations like these show the importance of a physician's clinical judgment in guiding management. Here, we would like to discuss a case where an asymptomatic 63-year-old man presented to the emergency department following a fall, but during the traumatic workup, an incidental lung mass was noted. Given the patient's lack of presenting hemoptysis, shortness of breath, unintentional weight loss, etc., with subsequent negative biopsy results, the patient was unconcerned regarding the pulmonary mass. Despite multiple attempts to contact the patient, he was lost to follow-up and returned with severe worsening of his malignancy. Thus, in this case report, we would like to discuss pulmonary sarcomatoid carcinoma, the methods of biopsy results, and the rate of false negatives, as well as highlight the importance of physicians' management and patient follow-up.

## Introduction

Pulmonary sarcomatoid carcinoma is a rare and aggressive subtype of non-small cell lung cancer, accounting for 0.1-0.4% of all pulmonary malignancies [1,2]. In 2021, the World Health Organization classified pulmonary sarcomatoid carcinoma into five subtypes: pleomorphic carcinoma, carcinosarcoma, giant cell carcinoma, spindle cell carcinoma, and pulmonary blastoma [2]. As such, due to pulmonary sarcomatoid carcinoma being diagnosed at an advanced stage, the disease carries a poor prognosis, with a five-year survival rate of only 15% [2,3].

Diagnosing pulmonary sarcomatoid carcinoma in and of itself is particularly challenging. Its histopathological heterogeneity often results in sampling error during biopsy, especially when necrosis, hemorrhage, or nonrepresentative tissue is obtained. Peripheral lung masses are typically obtained through a variety of techniques, including bronchoscopy, computed tomography (CT)-guided needle biopsy, and video-assisted thoracoscopic surgery [4-6]. CT-guided transthoracic needle biopsies (TTNB) are highly accurate in most lung malignancies, but carry a false-negative rate ranging from 4.6% to 16.4% [4]. Larger tumors are more challenging to diagnose, as necrosis and surrounding inflammation can obscure malignant tissue [5]. Likewise, bronchoscopic biopsies demonstrate variable sensitivity, from 36% to 88%, and may fail to capture diagnostically adequate tissue [6]. As a result, pulmonary sarcomatoid carcinoma can be commonly missed, even with multiple sampling attempts, leading to delays in diagnosis and treatment. Despite this, when there is a high suspicion of pulmonary malignancy, further workups should be done, and the patients should be informed accordingly regarding the possibility of false negatives.

Here, we present a case of pulmonary sarcomatoid carcinoma that was missed in two separate biopsy attempts, first on CT-guided core biopsy and subsequently on bronchoscopy, before finally being confirmed on a repeat needle biopsy. Unfortunately, the patient was lost to follow-up despite repeated attempts to contact the patient. As such, this case not only underlines the diagnostic challenges of pulmonary sarcomatoid carcinoma, notably the risk of false-negative biopsy results in large necrotic tumors, but also necessitates the importance of patient follow-up and medical compliance, which is especially true when imaging paints a concerning picture.

## Case presentation

**Figure 1:**
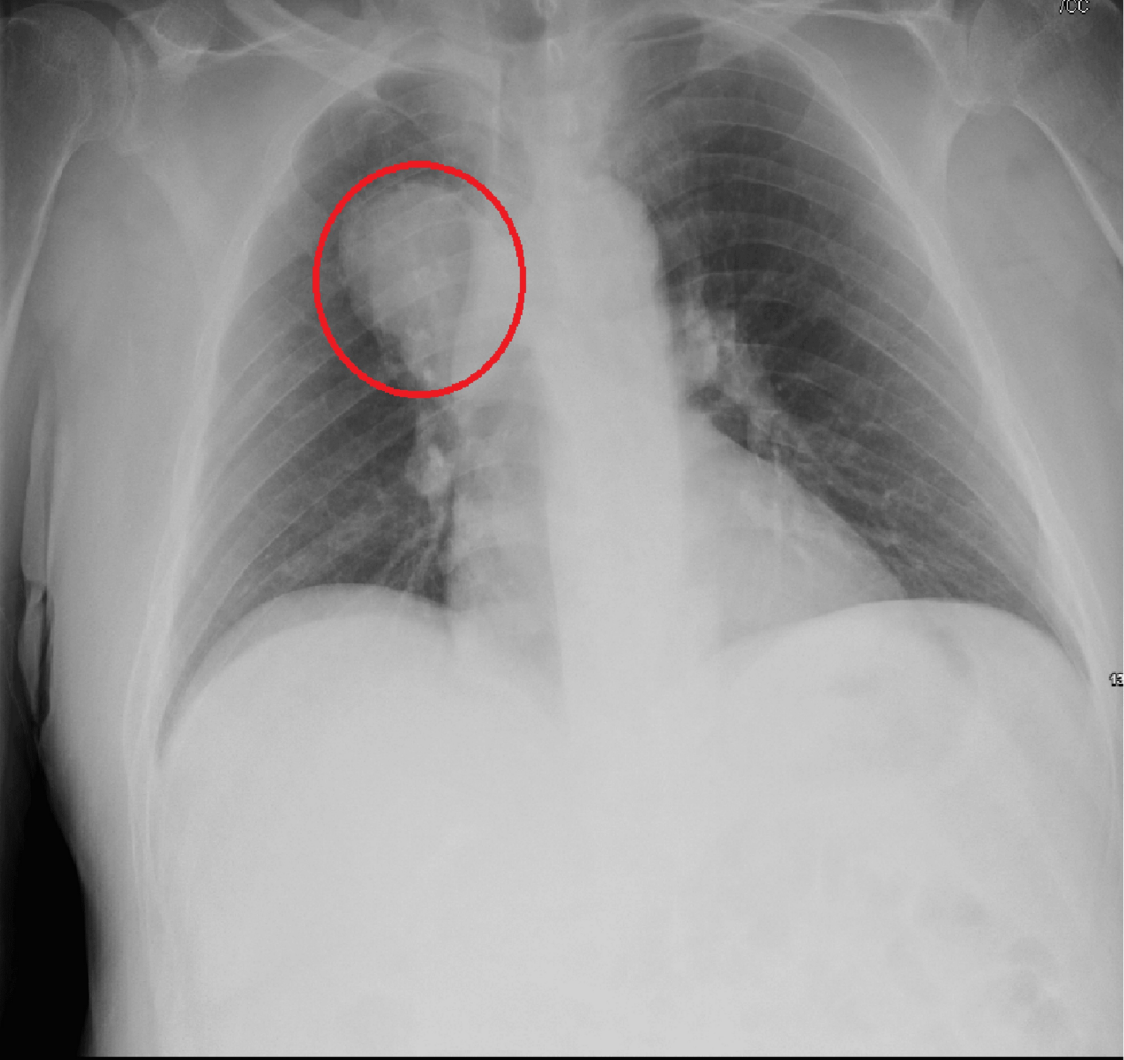
Initial chest X-ray There is a large indeterminate approximately 8-cm right perihilar/suprahilar mass indicated by the red circle; otherwise, the heart appears to be of normal size, and the lungs are clear, with no pneumothorax or significant pleural effusion noted.

**Figure 2:**
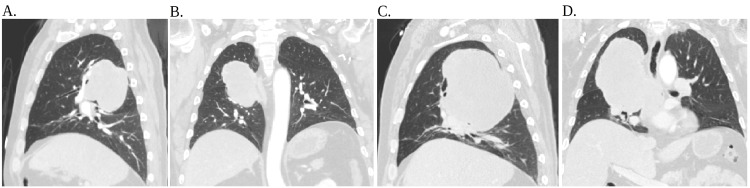
CT scan of the chest, abdomen, and pelvis (A) CT of the chest with contrast sagittal view shows a large right upper lobe/lower lobe pulmonary mass measuring 8×6×7 cm concerning for primary lung neoplasm. (B) CT of the chest with contrast transverse view shows a large right upper lobe/lower lobe pulmonary mass measuring 8×6×7 cm concerning for primary lung neoplasm. (C) CT of the chest with contrast sagittal view shows a pre-existing mass at the posterior right upper and mid-lung zone with contiguous posterior mediastinal extension which increased in size at a visit approximately one year later, now measuring 11×10×13 cm. (D) CT of the chest with contrast transverse view shows a pre-existing mass at the posterior right upper and mid-lung zone with contiguous posterior mediastinal extension which increased in size at a visit approximately one year later, now measuring 11×10×13 cm. CT: computed tomography

This patient is a 63-year-old man who initially presented to the emergency department after sustaining a 10-foot fall from his roof. A chest X-ray incidentally showed a large right upper lung mass, concerning for malignancy (Figure 1). He stated he had left hip pain but denied any headaches, fevers, chest pain, shortness of breath, or any other symptoms at the time. Vital signs and his complete blood count and basic metabolic panel were unremarkable (Table 1). The patient underwent whole-body CT, which showed a left acetabulum fracture, and the CT scan of the chest, abdomen, and pelvis (Figure 2A-2B) showed an 8-cm mass in the right upper lobe. The patient was admitted to medicine for further workup regarding the pulmonary mass while receiving orthopedic surgical intervention for his left acetabulum fracture. A CT-guided core biopsy was performed, and he was then discharged in stable condition to follow up on the pathology results in the outpatient clinic.

**Table 1:** Initial blood work

Parameter	Value	Reference range
Sodium	141 mmol/L	136-145 mmol/L
Potassium	4.6 mmol/L	3.5-5.1 mmol/L
Chloride	108 mmol/L	98-107 mmol/L
Calcium (corrected)	8.8 mg/dL	8.5-10.1 mg/dL
Magnesium	2.0 mg/dL	1.8-2.4 mg/dL
Phosphorus	3.5 mg/dL	2.5-4.9 mg/dL
Alanine transaminase	19 units/L	13-61 units/L
Aspartate transaminase	23 units/L	15-37 units/L
Alkaline phosphatase	109 units/L	45-117 units/L
Direct bilirubin	0.1 mg/dL	0-0.2 mg/dL
Total bilirubin	0.7 mg/dL	0-1 mg/dL
Albumin	3.1 g/dL	3.4-5 g/dL
White blood count (WBC)	16.8×10^3^/mcL	4.5-11×10^3^/mcL
Hemoglobin (Hgb)	13.8 g/dL	13.2-17.4 g/dL
Mean corpuscular value (MCV)	96.5 HI	80-98 HI
Platelet	374×10^3^/mcL	150-450×10^3^/mcL
Neutrophil %	87.2%	50-75%
Lymphocyte %	6%	20-45%
Bands %	0%	<12%
Monocyte %	6.6%	2-12%
Eosinophil %	0.1%	<6%
Absolute neutrophil	14.7×10^3^/mcL	1.8-7.7×10^3^/mcL
Absolute lymphocyte	1.0×10^3^/mcL	1.2-4.5×10^3^/mcL
Absolute monocyte	1.1×10^3^/mcL	0.1-1×10^3^/mcL
Absolute eosinophil	0×10^3^/mcL	<0.7×10^3^/mcL

The following week at the outpatient clinic, further history was obtained. He endorsed only mild to moderate localized left hip pain but otherwise denied any other symptoms. He admitted to some weight loss but attributed it to dieting. The patient stated that his medical history was limited to benign prostatic hyperplasia, for which he was taking tamsulosin 0.4 mg daily. He was taking medications prescribed to him for his hip fracture and denied any surgical history besides the recent open reduction internal fixation of his left acetabulum. The rest of his history, including family history, was unremarkable. His employment history included five years at a chemical plant producing pesticides and several years at a casino, where he was exposed to secondhand smoke. He later transitioned to working as a cook and is now employed as a roofer in his current role. The patient reported drinking 1-2 beers on the weekends but denied any tobacco use or any other recreational drug use, including marijuana, cocaine, amphetamines, etc.

**Figure 3:**
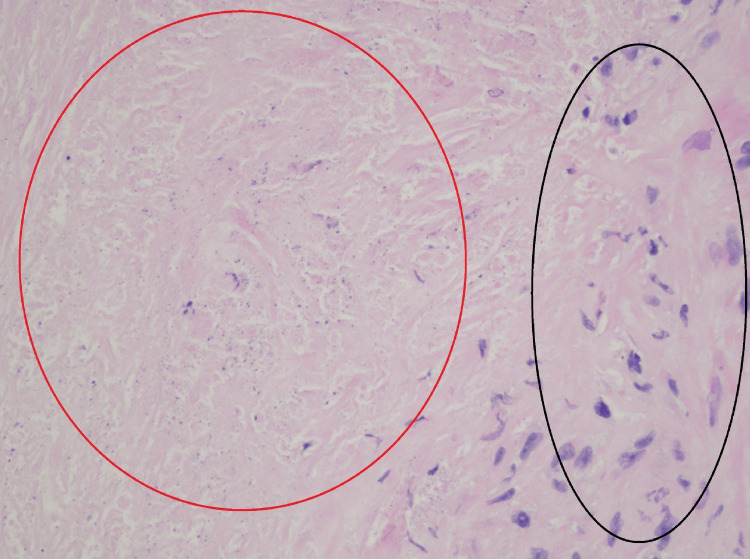
Initial lung biopsy results with hematoxylin and eosin stain The red circle indicates areas of necrosis, whereas the black circle indicates the granuloma with spindle cells noted overall. Necrotizing granulomatous inflammation is seen. Periodic acid-Schiff and methenamine silver stain are negative for fungal organisms, while acid-fast bacilli stain is negative for acid-fast bacilli.

On physical examination, he had a normal blood pressure of 120/75, was afebrile, had a normal heart rate and respiratory rate, and was saturating well in room air. The physical exam revealed mildly decreased breath sounds, specifically in the posterior right portion of the patient's upper lung. Additionally, dullness to percussion was noted over the posterior right upper lung field, accompanied by increased tactile fremitus and egophony in the same area. No crackles, rales, wheezing, or rhonchi were appreciated. The remainder of the physical exam was unremarkable. The patient was then informed of his negative pathology results (Figure 3). At the current time, a differential diagnosis was quite broad, including benign tumors such as hamartomas versus infections such as fungal infections versus inflammatory conditions such as sarcoidosis versus malignancy. However, malignancy was high on the differential diagnosis despite the negative biopsy results. He was further informed that, regardless of the biopsy results showing no malignancy, a repeat biopsy and further workup were needed for further evaluation due to the concern of false-negative biopsy results. Unfortunately, the patient was transiently lost to follow-up despite numerous attempts to contact the patient via email, telephone, and mail.

**Figure 4:**
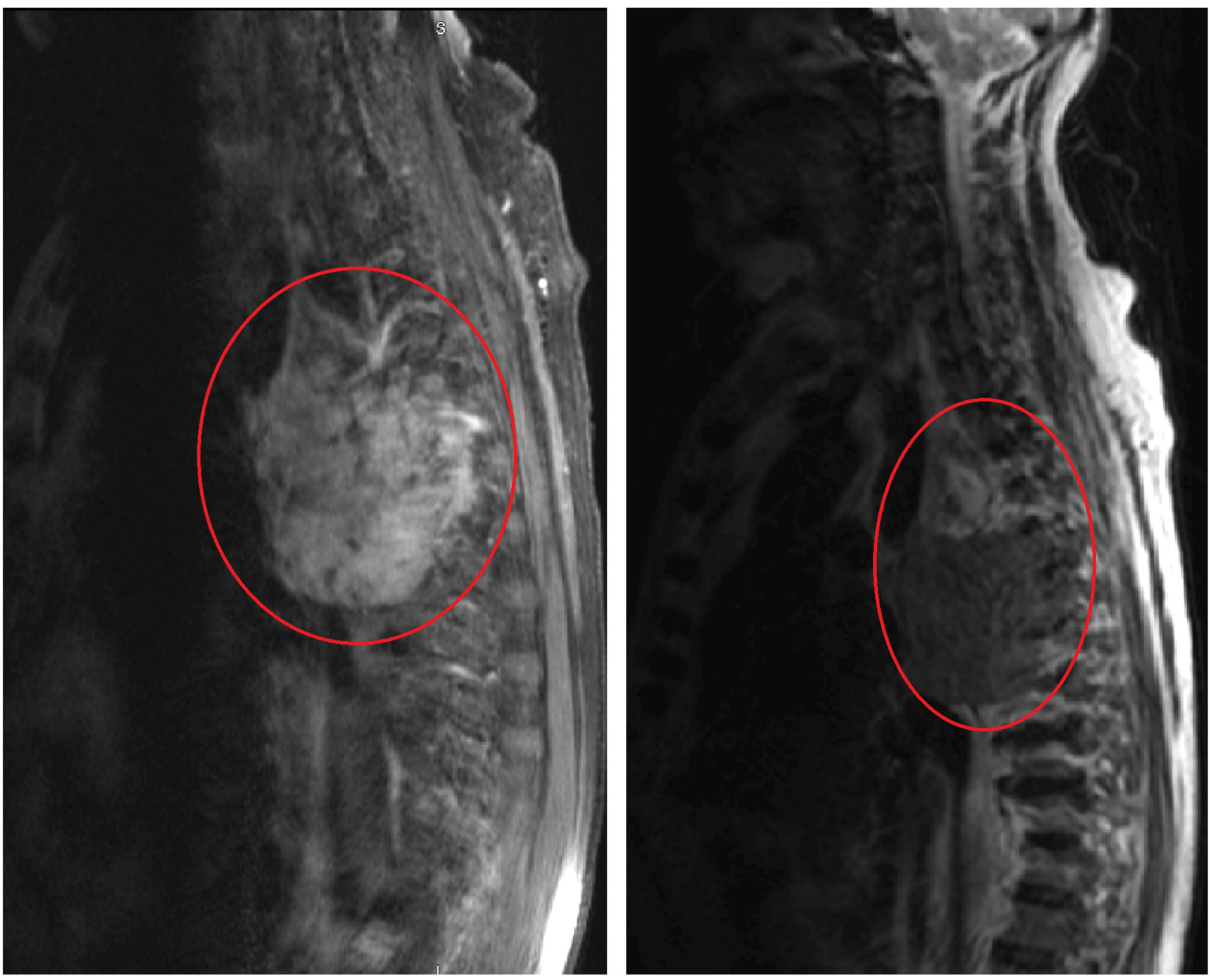
MRI of the thoracic spine with and without contrast The large mass indicated by the red circle is located in the right hemithorax encroaching on the right neural foramina at T5-T6 and T6-T7, contributing to severe neural foraminal stenosis at T5-T6 on the right. No significant spinal canal stenosis. MRI: magnetic resonance imaging

**Figure 5:**
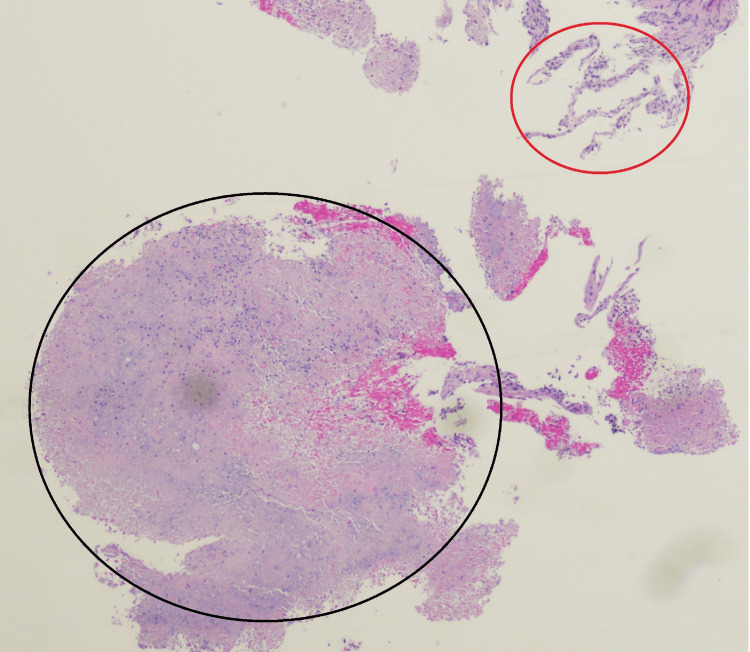
Transbronchial biopsy with hematoxylin and eosin stain The black circle shows the area of necrotic debris and the red circle shows the lung parenchyma; the rest of the transbronchial biopsy of the right lung shows the lung parenchyma with fibrosis, hemorrhage, acute inflammation, and necrosis. Periodic acid-Schiff and methenamine silver stain are negative for fungal organisms, and acid-fast bacilli stain is negative for acid-fast bacilli, while the rest of the biopsy results are negative for malignancy. Bronchial brush smear shows many red blood cells with admixed respiratory epithelial cells, macrophage, and a few squamous cells with focal acute inflammation with no evidence of malignancy. Bronchial brush tip shows small fragments of parenchyma with focal squamous metaplasia, focal acute inflammation, and focal necrotic debris, no evidence of acid fast or fungal organisms, and no evidence of malignancy. Bronchoalveolar lavage of the right upper lobe shows mostly macrophages with no evidence of malignancy. Bronchial wash of the right upper lobe fluid shows admixed respiratory and squamous epithelial cells with macrophages with no evidence of malignancy.

One year later, he returned to the emergency department due to progressive shortness of breath and chest pain for 2-3 weeks. He denied fever, chills, cough, hemoptysis, dysphagia, or hoarseness. A repeat CT scan of the chest showed that the pulmonary mass had increased in size (Figure 2C-2D). The patient was admitted, and pulmonology and oncology were consulted, with further blood work done (Table 2). Magnetic resonance imaging (MRI) of the thoracic spine was ordered to evaluate for potential malignant extension, and findings indicated a large mass in the right hemithorax encroaching on the right neural foramina (Figure 4). He underwent bronchoscopy; however, there was difficulty advancing the scope to the narrowed area of interest of the posterior segment of the right upper lobe. Bronchial alveolar lavage (BAL) was done from the anterior segment of the right upper lobe. Five transbronchial biopsies (Figure 5) from the posterior segment of the right upper lobe were obtained. Again, pathology results showed acute inflammation and necrosis and were negative for malignancy.

**Table 2:** Further blood work CF: complement fixation; ANCA: anti-neutrophil cytoplasmic antibodies; C-ANCA: cytoplasmic anti-neutrophil cytoplasmic antibodies; P-ANCA: perinuclear anti-neutrophil cytoplasmic antibodies; CCP: cyclic citrullinated peptide

Parameter	Value	Reference range
Coccidioidomycosis IgM	Nonreactive	Nonreactive
Coccidioidomycosis IgG	Nonreactive	Nonreactive
Coccidioidomycosis CF antibody	Nonreactive	Nonreactive
Acid-fast bacteria smear	Negative for acid-fast bacteria	Negative
Mycobacteria culture	No mycobacterium	Negative
Immunoglobulin E	102 kU/L	<114 kU/L
ANCA	Negative	Negative
C-ANCA	Not detected	<1:20
P-ANCA	Not detected	<1:20
Cryptococcal antigen	Not detected	Not detected
Echinococcus antibody IgG	Not detected	Not detected
CCP antibody IgG	<16 units	<20 units
IgG subclass 4	82.6 mg/dL	4-86 mg/dL
QuantiFERON-TB Gold	Negative	Negative

**Figure 6:**
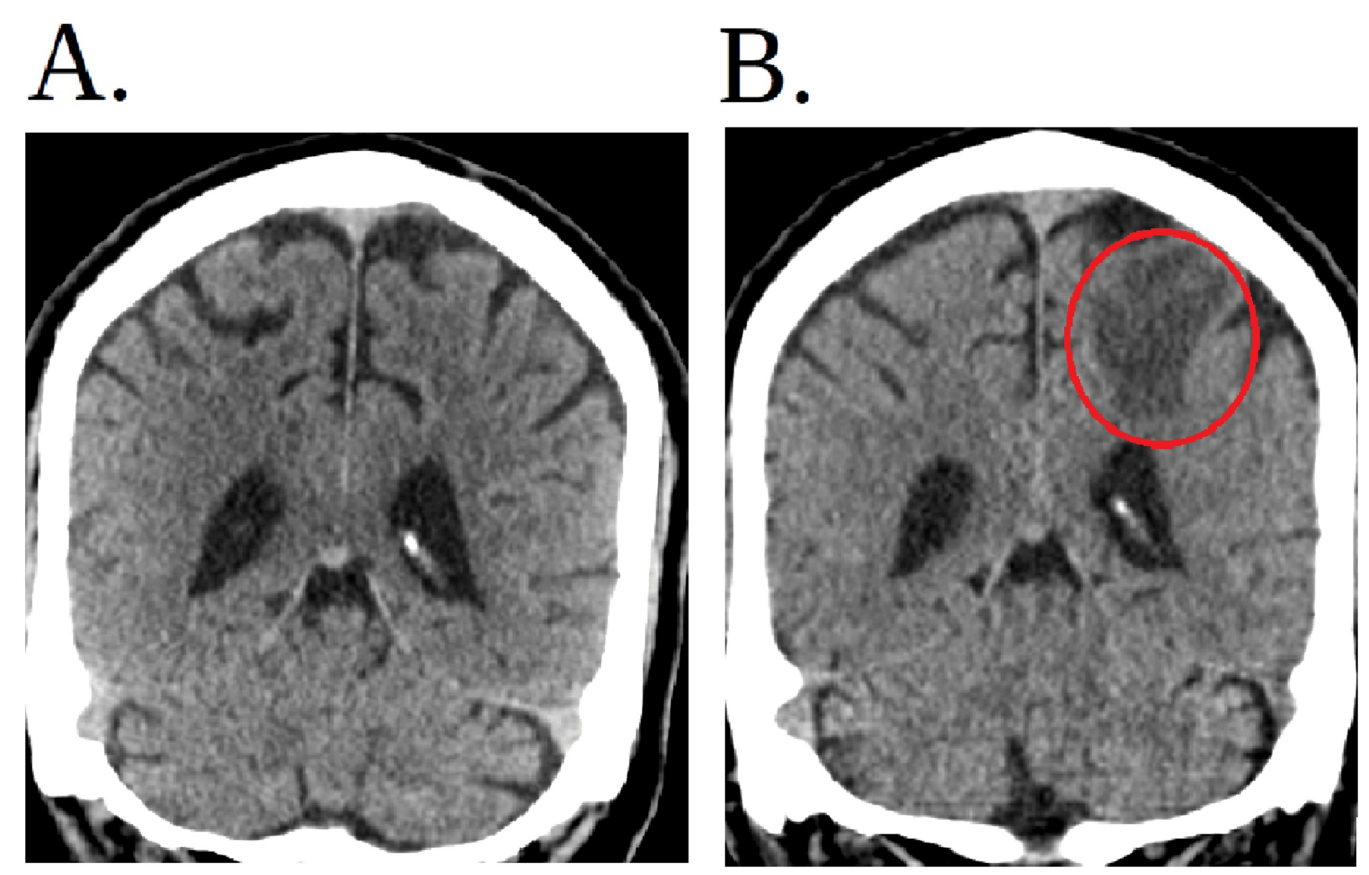
CT of the brain and head (A) Initial CT scan that was done in the emergency department at first presentation when the patient had his trauma from falling from his roof which shows no metastasis. (B) Repeat CT of the brain/head without contrast approximately one year later shows probable left parietal lobe intra-axial metastasis with associated vasogenic edema noted, new since the initial CT scan of the brain. CT: computed tomography

**Figure 7:**
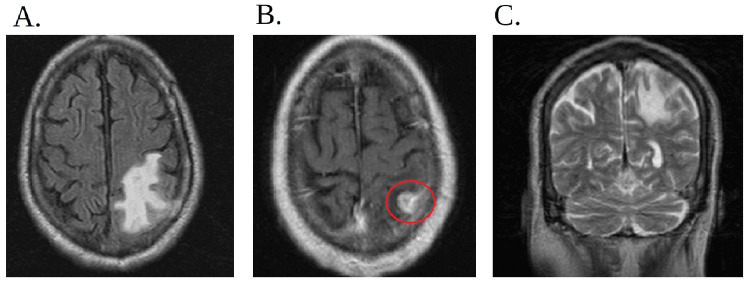
MRI of the brain and head with and without contrast (A) The figure is a transverse view of the MRI of the brain with and without contrast which shows prominent adjacent vasogenic edema. (B) The red circle indicates a 1.5-cm left parietal lobe extra-axial enhancing mass. (C) The figure is a coronal view of the MRI of the brain with and without contrast which shows prominent adjacent vasogenic edema in the left parietal lobe. MRI: magnetic resonance imaging

Shortly after, the patient developed seizures. A CT of the brain and head showed a left parietal lobe intra-axial metastasis with associated vasogenic edema, suspicious for malignancy, with an MRI of the brain and head with and without contrast done for further evaluation (Figure 6A-6B and Figure 7A-7C). The patient was managed with a one-time dose of intravenous dexamethasone 10 mg followed by 4 mg every six hours, as well as levetiracetam 1000 mg every 12 hours. A third biopsy, now a CT-guided needle lung biopsy, was performed due to the high suspicion of malignancy based on the recommendations from hematology and oncology, who believed that the patient had suffered a brain metastasis most likely from lung cancer.

**Figure 8:**
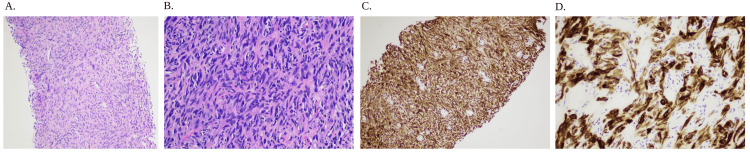
CT-guided lung biopsy path results (A) This is a ×10 magnification of a hematoxylin and eosin stain with prominent spindle cells. (B) This is a ×20 magnification of a hematoxylin and eosin stain with prominent spindle cells. (C) This is a ×10 magnification of a cytokeratin staining with the brown showing positive for cytokeratin AE1/AE3. The strong expression of cytokeratin AE1/AE3 in the neoplastic cells is consistent with a sarcomatoid (spindle cell) carcinoma. (D) This is a ×20 magnification of a cytokeratin staining with the brown showing positive for cytokeratin AE1/AE3. The strong expression of cytokeratin AE1/AE3 in the neoplastic cells is consistent with a sarcomatoid (spindle cell) carcinoma. Staining were as follows: cytokeratin AE1/AE3, positive; SMA, focally positive; STAT6, negative; desmin, negative; CD34, negative; CD117, negative; S100, negative; and TLE1, negative. The strong expression of cytokeratin AE1/AE3 in the neoplastic cells is consistent with a sarcomatoid (spindle cell) carcinoma. No other heterologous foci of differentiation are seen in these biopsies. The focal co-expression of smooth muscle actin is of uncertain significance. There is no immunohistochemical evidence of solitary fibrous tumor, leiomyosarcoma, rhabdomyosarcoma, synovial sarcoma, liposarcoma, or melanoma. CT: computed tomography

Neurosurgery was consulted and agreed that the patient had primary lung cancer that had metastasized to the brain and noted that no neurosurgical intervention is presently indicated. An MRI of the lumbar spine without contrast was done, which showed no evidence of metastatic disease to the lumbar spine. The patient improved on the dexamethasone and levetiracetam, was observed in the hospital with no further occurrence of seizures, and was observed to be alert, interactive, and oriented to self, date, and place. He was then educated regarding the extreme importance of follow-up and was discharged with neurology, oncology, and primary care physician follow-up appointments. He was then informed of the follow-up appointments regarding the repeat path results the following week. Repeat pathology results confirmed sarcomatoid carcinoma (Figure 8). However, again, despite multiple attempts to contact the patient, he did not appear for the scheduled follow-up.

**Figure 9:**
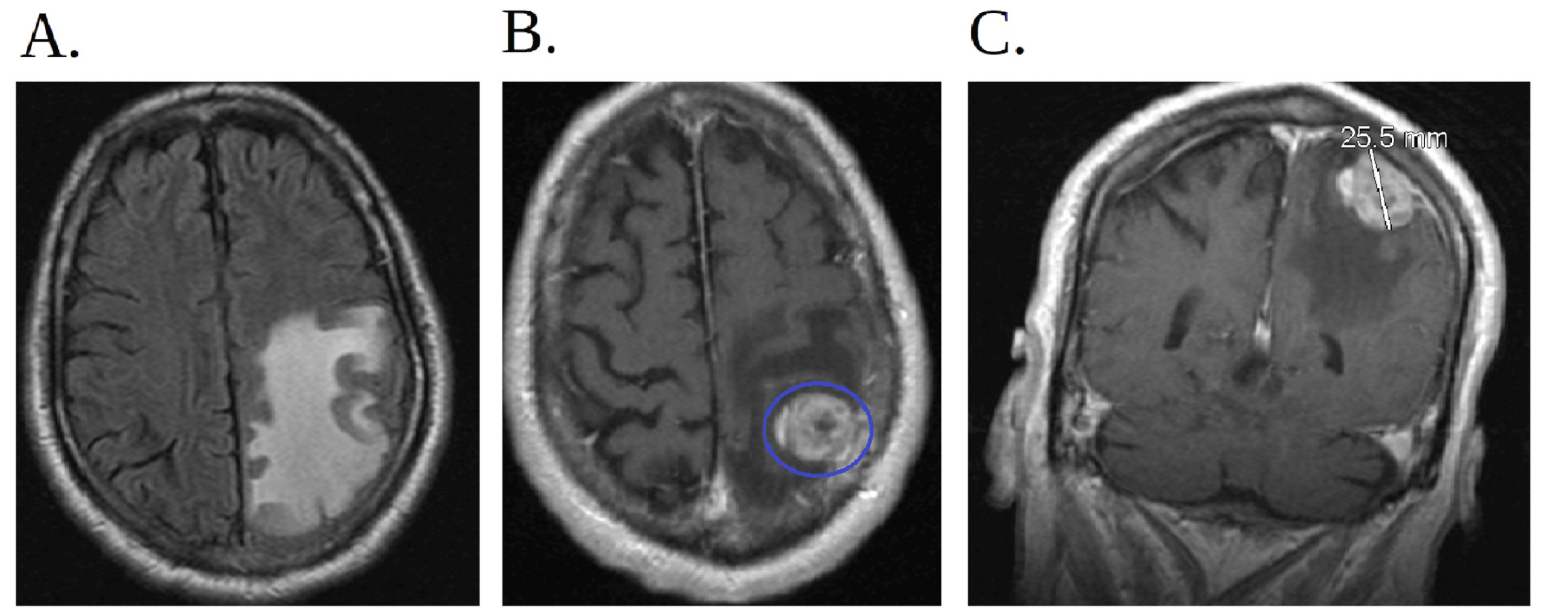
Repeat MRI of the brain and head with and without contrast (A) The figure is a transverse view of the MRI of the brain with and without contrast which was done two months later and shows worsening prominent adjacent vasogenic edema. (B) The blue circle indicates an increasing size of a left parietal cortical/subcortical mass now measuring 2.6×1.9×2.2 cm with increasing vasogenic edema involving the left frontal and parietal lobes which was done two months later from the initial MRI of the brain. (C) The figure is a coronal view of the MRI of the brain with and without contrast which was done two months later from the first MRI of the brain and shows the coronal view of the left parietal cortical/subcortical mass now measuring 2.6×1.9×2.2 cm. MRI: magnetic resonance imaging

**Figure 10:**
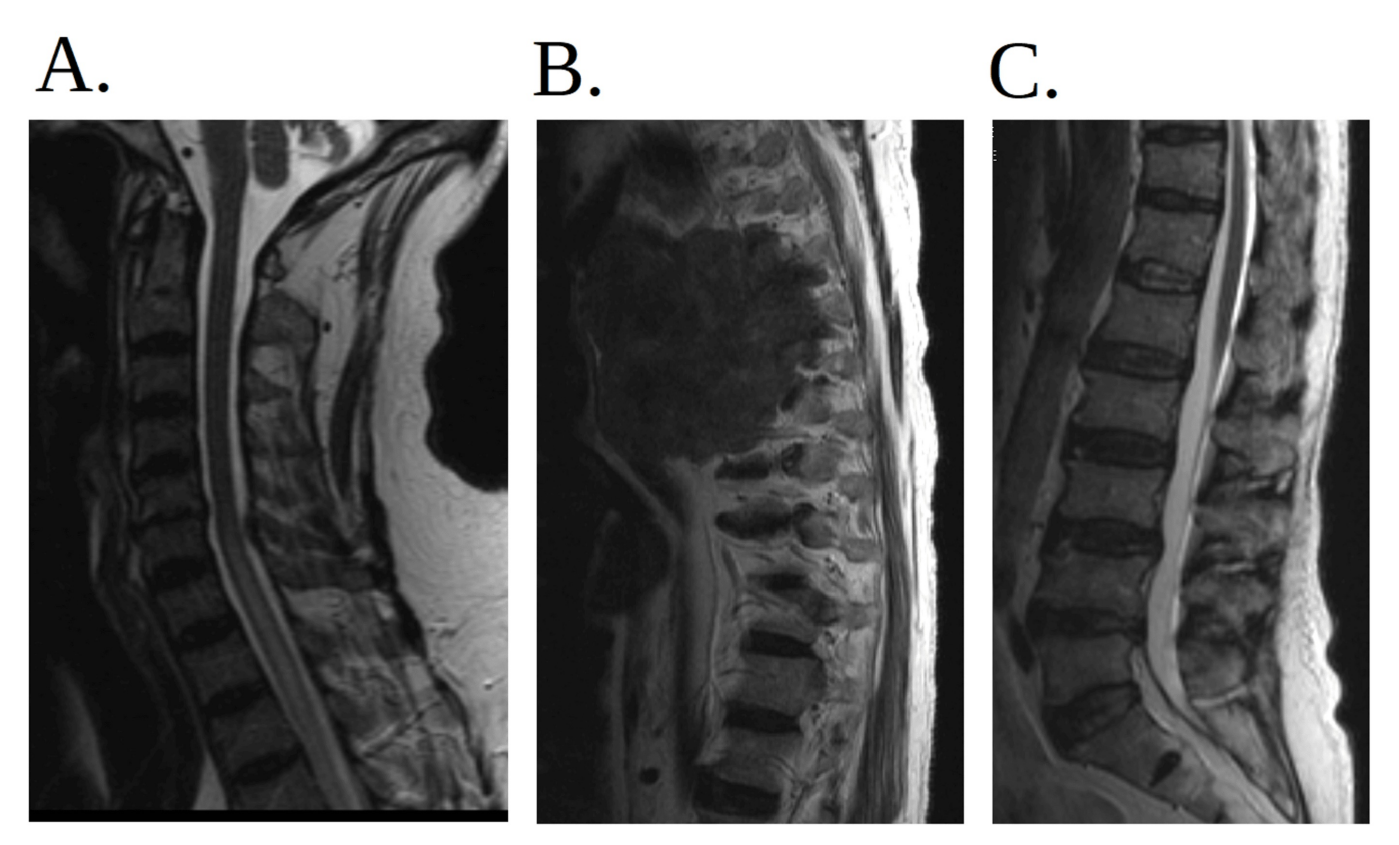
MRI of the cervical, thoracic, and lumbar spine (A) MRI of the cervical spine shows partially included syrinx extending from the thoracic spine up to the C5 level with no definitive focal cervical spinal cord lesion or compression. (B) MRI of the thoracic spine shows that the mass invades the T4-T7 vertebral bodies with pathologic fracture of the T6 vertebral body and the pulmonary mass extends through the right T5-T6 and T6-T7 neural foramen into the spinal canal, abutting/invading the spinal cord with enhancement of the cord from T4 to T7. There are nodular soft tissue densities extending into the central canal at the T4-T5 level with the development of a syrinx extending from the C5 to T9 vertebral levels. (C) MRI of the lumbar spine shows no metastatic lesions within the lumbar spine and mild spondylosis with moderate to severe bilateral neural foraminal narrowing at L4-L5. MRI: magnetic resonance imaging

The patient returned to the emergency department two months later due to multiple falls at home. MRI of the brain (Figure 9A-9C) showed an increasing size of a left parietal cortical/subcortical mass with increasing vasogenic edema involving the left frontal and parietal lobes. Again, the patient was treated with dexamethasone and levetiracetam. Due to his neurological findings, an MRI of the cervical, thoracic, and lumbar spine was done, which showed that the right upper lobe mass had invaded the T4-T7 vertebral bodies with pathologic fracture of the T6 vertebral body (Figure 10A-10C). Neurosurgery recommended transfer to a higher level of care for multidisciplinary management. Unfortunately, the patient quickly deteriorated, and both the patient and the family elected for a do not resuscitate/do not intubate code status. The patient and his family members had a discussion with the palliative team, who deemed him appropriate for outpatient follow-up with the option to transition to hospice care in the future, to which both the patient and family members agreed. He was discharged accordingly and passed away a few weeks later.

## Discussion

Pulmonary nodules and masses have their own management according to various general consensus guidelines [7-9]. One study noted that approximately 95% of all pulmonary nodules are benign in nature [7]. The same study also noted that nodules with a size of <6 mm have a less than 1% chance of being malignant, while nodules measuring 6-8 mm have a 1-2% chance of being malignant [7]. When discovered nodules are >8 mm in size, various risk factors and the patient history play a crucial part in determining the risk of malignancy, with management ranging from repeat CT imaging after three months vs. a biopsy and/or resection [7-9]. This is especially true when the etiology of pulmonary nodules ranges from infection to inflammatory lesions to benign masses such as pulmonary hamartoma, etc. Despite this, there are rare instances of repeatedly negative biopsy results that fail to ascertain a proper diagnosis [4-6]. This is especially the case when examining the false‑negative rate of CT‑guided TTNB. CT‑guided TTNB has become the most used diagnostic method for evaluating brain masses due to its high accuracy and low rate of complications. TTNB has a sensitivity of 85.7-97.4%, a specificity of 88.6-100%, and an accuracy of 89-96.9% [4]. Unfortunately, the false‑negative rate of the procedure ranges from 4.6% to 16.4%, with no clear consensus on next steps in response to non‑specific benign pathological results [4]. As such, the burden of this decision rests on the individual clinician and their index of suspicion.

Cultivating this decision-making is key, especially since pulmonary sarcomatoid carcinoma metastases commonly involve organs such as the lung, adrenal glands, pleura, brain, bone, or liver. These tumors may also metastasize to unusual anatomic sites, such as subdiaphragmatic nodes, kidney, peritoneum, pancreas, skin, and right ventricle [1]. Older age, pleural traction, and burr sign presence are all important risk factors for false‑negative results, especially in conjunction with other independent risk factors such as surgeon experience, lower lobe lesions, tumor size, etc. [4]. Interestingly, larger tumors have been associated with a higher incidence of false-negative results. This is because of the increased difficulty in differentiating between the primary malignant lesion and the surrounding inflammation or necrosis [5].

Other diagnostic methods aimed at increasing rates of true-positive biopsy results have also been utilized. Bronchoscopy, for example, has an accuracy of approximately 36-88% for the diagnosis of peripheral pulmonary malignancy. Additionally, peripheral transbronchial needle aspiration (TBNA) is also known to provide better accuracy; however, it can be limited in the assessment of lesions in the upper lobes and superior segments of the lower lobes, where the need for sharp turns can prevent adequate access of the straight needle [6]. Previous studies have also reported no increase in mortality when diagnosis is delayed after a false‑negative result as long as the patient with an indeterminate lesion is closely monitored over time, which proved to be the major limiting factor in our patient's case. Nevertheless, this case demonstrates the vital role of clinician judgment when pulmonary masses present with suspicious traits, in this case, size and spread.

The large size of the patient's pulmonary mass indicated a high suspicion of malignancy. However, the patient's asymptomatic presentation and unremarkable medical history were unique for a neoplastic process, additionally complicated by two negative biopsies. Despite these initial findings, the mass progressively developed in size, and a physician's consideration of this growth activity contributed crucially to the decision to continue sampling the mass despite previous negative results. The rapid development of the mass also caused many of the symptoms that the patient eventually developed over the course of his disease. 

Due to the concerns manifested by the size and the growth of the mass, the patient was informed of the necessity of further management and close observation via repeated CT imaging at a minimum, even with an initially negative biopsy result [7]. However, since the patient had no symptoms and no significant risk factors for malignancy at his initial outpatient follow-up visit, he himself was unconcerned and was lost to follow-up.

Unfortunately, when the patient started developing symptoms, the malignancy had progressed, and a diagnosis of pulmonary sarcomatoid carcinoma was not made until the third pathology result. As a rare and highly aggressive lung malignancy, pulmonary sarcomatoid carcinoma should be treated immediately. Unfortunately, the clinical presentations, such as dyspnea, cough, hemoptysis, chest pain, and weight loss, are non-specific. Thus, diagnosing the disease itself is difficult. The median age at diagnosis is 68 years, and the median tumor size is 5 cm, with the affected patient demographic more commonly males with a history of smoking [3]. On imaging, CT characteristics include commonly peripheral tumors, upper lobe lesions, and round or circular masses with well-defined borders, and on occasion, subpleural lesions tend to invade the chest wall or pleura [1]. However, when there are concerns of any lung malignancy, biopsy results should be obtained to help guide management, but as stated before, false negatives can occur. Due to the aggressive nature of this disease, the reported median survival is about six months, while the five-year survival rates are estimated to be around 15%, with approximately 62% of patients having more than two metastatic locations [3]. As such, a physician's judgment regarding close monitoring and frequent biopsies should play an important role in ensuring proper medical care.

## Conclusions

This case overwhelmingly implicates the importance of a strong clinical suspicion in the context of a large lung mass, despite multiple negative biopsy results in an asymptomatic patient. Patients should be informed accordingly about the likelihood of false negatives and should have close monitoring and follow-up due to the concerns of a rapidly malignant cancer in worst-case scenarios such as this. Hopefully, this case provides both patients and physicians with awareness that, despite a lack of symptoms and negative biopsy results, alarming imaging should not be ignored and should continue to serve as a crucial tool in management.
